# Domains of quality for clinical ethics case consultation: a mixed-method systematic review

**DOI:** 10.1186/s13643-016-0273-x

**Published:** 2016-06-07

**Authors:** Louis Leslie, Rebecca Frances Cherry, Abbas Mulla, Jean Abbott, Kristin Furfari, Jacqueline J. Glover, Benjamin Harnke, Matthew K. Wynia

**Affiliations:** School of Medicine, University of Colorado, Anschutz Medical Campus, Denver, CO USA; University of Colorado at Denver, Downtown Campus, Denver, CO USA; Center for Bioethics and Humanities, University of Colorado, Anschutz Medical Campus, Denver, CO USA; Health Sciences Library, University of Colorado, Anschutz Medical Campus, Denver, CO USA; University of Colorado-Center for Bioethics & Humanities, Fulginiti Pavilion for Bioethics and Humanities, Mailstop B137, 13080 E. 19th Avenue, Aurora, CO 80045 USA

**Keywords:** Clinical ethics, Health care ethics committees, Ethics consultation, Mixed-method, Scoping review, Bioethics, Systematic review

## Abstract

**Background:**

“Clinical ethics consultation” (CEC) is the provision of consultative services by an individual or team with the aim of helping health professionals, patients, and their families grapple with difficult ethical issues arising during health care. There are almost 25,000 articles in the worldwide literature on CEC, but very few explicitly address measuring the quality of CEC. Many more address quality implicitly, however. This article describes a rigorous protocol for compiling the diverse literature on CEC, analyzing it with a quality measurement lens, and seeking a set of potential quality domains for CEC based on areas of existing, but hitherto unrecognized, consensus in the literature.

**Methods/design:**

This mixed-method systematic review will follow a sequential pattern: scoping review, qualitative synthesis, and then a quantitative synthesis. The scoping review will include categorizing all quality measures for CEC discussed in the literature, both quantitative and qualitative. The qualitative synthesis will generate a comprehensive analytic framework for understanding the quality of CEC and is expected to inform the quantitative synthesis, which will be a meta-analysis of studies reporting the effects of CEC on pre-specified clinical outcomes.

**Discussion:**

The literature on CEC is broad and diverse and has never been examined with specific regard to quality measurement. We propose a novel mixed-methods approach to compile and synthesize this literature and to derive a framework for assessing quality in CEC.

**Systematic review registration:**

PROSPERO CRD42015023282

**Electronic supplementary material:**

The online version of this article (doi:10.1186/s13643-016-0273-x) contains supplementary material, which is available to authorized users.

## Background

The ethics of health care delivery are famously complex. While classical principles of medical ethics such as beneficence, justice, and autonomy can provide some guidance, how are health professionals to decide when an action that may be beneficial conflicts with obligations to justice or to respecting patient autonomy? To help health professionals, patients, and their families navigate such challenging questions, the field of clinical ethics consultation was born [1, 2].

The American Society for Bioethics and Humanities (ASBH) defines clinical ethics consultation (CEC) as “a service provided by an individual or a group to help patients, families, surrogates, health care providers, or other involved parties to address uncertainty or conflict regarding value-laden issues that emerge in healthcare.” [2] These services have been part of most hospitals’ operations for more than 20 years [1]. In 1983, Younger et al. found that only 1 % of hospitals in the USA reported having an ethics committee [3], but by 1998, over 90 % of US hospitals had created ethics committees [4, 5]. This burgeoning growth was stimulated in part by a 1992 requirement of The Joint Commission (TJC) that hospitals develop a “mechanism” to help teams address ethical dilemmas in the care of hospitalized patients [6].

This rapid increase in clinical ethics committees, both in the USA and abroad, has naturally led to discussions about how to define and measure the quality of the CEC services they provide [7–15]. Much debate has ensued about how, or even if, CEC services can be evaluated and what elements are most critical [16].

To develop tools to measure quality of CEC, an analytical model must first be developed that describes the basic aims of these consultations. Creating such a model de novo can be thought of as analogous to developing a new survey tool: it should start with focus groups and structured interviews in the community of interest, to craft the language and concepts that the survey tool will eventually try to measure. Similarly, to better understand the common language and concepts of quality in CEC, we propose to use the broad field of literature related to CEC as our community information source, with qualitative and quantitative analysis of this literature as analogues to focus groups and structured interviews.

To our knowledge, no formal systematic review of the literature around CEC has been conducted that aimed to list possible CEC quality measures or to derive a set of general categories of such measures (i.e., CEC quality domains). We are therefore planning to carry out a structured, in-depth examination of the literature addressing CEC quality using a sequential mixed-method approach [17, 18], which will proceed in three phases as described below.

If we find reasonable levels of consensus within the literature around some of the quality measures or domains derived from the proposed approach, these might then serve as a starting point for the development of uniform quality measures for CEC services. Having a consensus set of CEC quality domains and measures might also contribute toward the continued evolution of CEC practice guidelines [19, 20] and could inform ongoing discussions about the professionalization of the field.

## Objectives

This review has three primary objectives:

1. List the quality measures of CEC found in the literature—scoping review
2. Develop an analytical structure for measuring the quality of CEC—qualitative synthesis
3. Assess the effects of CEC on clinical and other outcomes—quantitative synthesis

**Fig. 1:**
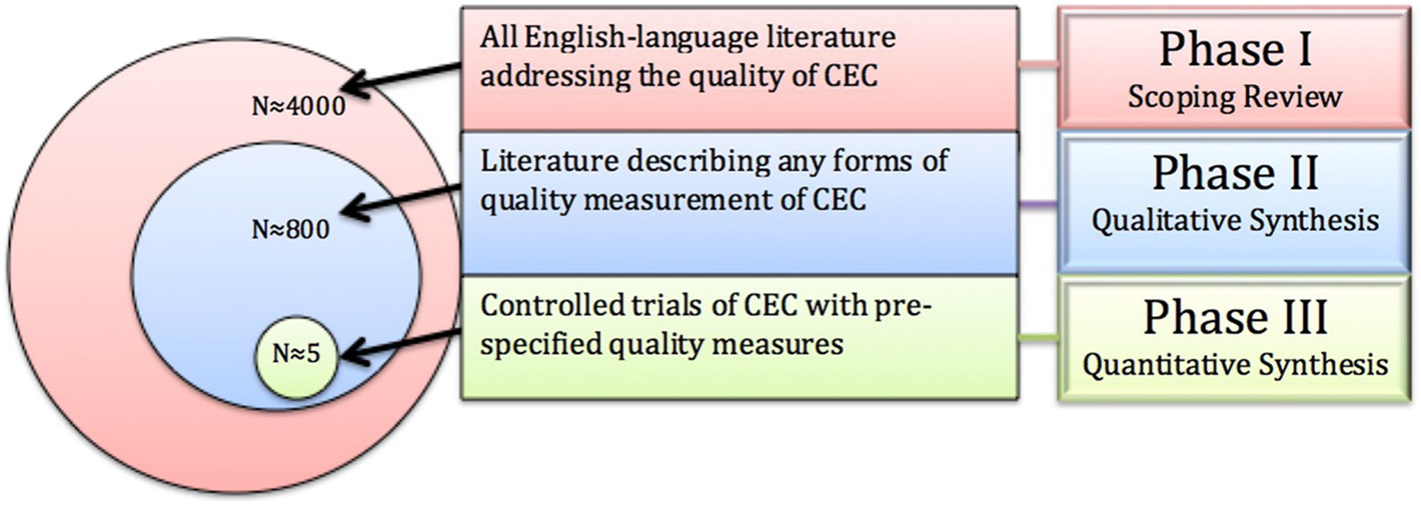
Summary of mixed-method review

Our initial probe of the literature found around 28,000 articles that touch on CEC. Based on an initial scan of titles and abstracts, we estimate that roughly 4000 are likely to include some specific discussion of quality and meet other inclusion criteria, as shown in Fig. 1. The figure also shows the basic outline of our structured, mixed-methods approach to this project.

Phase I will be a scoping review, using comprehensive literature review methods as detailed below [21]. In brief, we will use a rigorous search strategy and categorize to organize the literature on CEC as it pertains to quality measurement. This serves a two-fold purpose of (1) compiling the literature needed for the next two phases and (2) creating a preliminary list of potential quality measures that will be used to help organize our work in subsequent phases and which may also be useful for other researchers in this field.

Phase II will be a formal synthesis of the qualitative literature on CEC quality, looking for potentially measurable quality domains. This will follow the iterative approach of a thematic analysis [22, 23] where multiple ideas and conclusions across a body of literature are summarized into key themes, which are then refined into categories and sub-themes. It is these shared themes that might then provide an analytical structure for measuring the quality of CEC services.

Phase III will be a formal synthesis of studies assessing the effects of CEC on pre-specified outcomes. We expect this literature to be rather limited (Fig. 1), but we will use meta-analytic methods if possible. If there is not sufficient homogeneity in RCT methods and outcomes to perform a meta-analysis, then we will produce a narrative summary of the findings per standard practice [24, 25].

In a sequential mixed-method synthesis, the integration of qualitative and quantitative data typically occurs in the final phase [17, 18], which is phase III (quantitative synthesis) for our purposes. For example, the RCTs we have found in our preliminary search, which will be the subject of the phase III meta-analysis, have demonstrated generally positive findings for CEC, but there are pertinent study design differences that impact positive outcomes. We expect to illuminate these key factors using the derived analytical structure from phase II. Similarly, we hope to show that consultation efficacy (effect size of outcome measure, obtained in phase III) is a product of consultation quality (using new measures derived from the phase II qualitative synthesis).

## Methods

Because there are three methodologically distinct phases to this process, each will be described separately. As shown in Fig. 1, the phases are dependent on each other but the methods of each are unique. In particular, phase I will include the search function necessary for carrying out the subsequent qualitative and quantitative syntheses. As described below, data extraction, analysis, and findings will be reported separately for each part of this mixed-method synthesis.

### Phase I (scoping review) methods

The aim for phase I is to compile the entire published English language literature that relates directly to quality measurement of CEC and to categorize this literature into relevant study types, findings, and so on. This comprehensive scoping review of the literature will be performed following the steps outlined by Arksey et al. [26].

#### Study inclusion and exclusion criteria

We will include all studies that reference any quality measures—whether of structure, process, or outcomes—of CEC, whether implicitly or explicitly, and including but not limited to studies using the following methods: randomized-controlled trial, cohort, case-control, cross-sectional, survey studies, qualitative studies, case series, and non-research analytic papers. In short, studies will not be excluded on the basis of methodology or quality. Non-human studies will be excluded, as will studies that describe only other non-consultative activities of ethics committees (e.g., educational outreach, research ethics consultations, policy development, or organizational ethics work). Case studies and case series addressing only bioethical content of consults will be excluded, but case studies or series will be included if they address quality measurement related to structures, processes, or outcomes of ethics consultation. Non-research analytic articles will be included if they address quality measurement, since opinions in these articles, taken together, can provide guidance on how a complex intervention works, as has been shown in other types of literature review [22, 27].

#### Types of participants

Included studies may address or examine any individuals or groups involved in the ethics consultation process in any of the eligible literature. This may include patients, families, ethicists, health care workers, chaplains, lawyers, and other stakeholders [8].

#### Types of interventions

The intervention under review is an ethics consultation provided by an individual or team from an ethics consultation service in a hospital or other health care delivery organization. In the USA, the process of CEC usually includes a request for a consultation, assessment of that request, ethical workup, recommendations, and then documentation and potential follow-up [28]. This typical process, however, is widely variable. Ethics case consultation services can be provided by single individuals or multi-person committees, volunteers or professionals, and members of the community or employees, often with great variation in education and certification (and presumably, quality) [8, 29, 30]. Likewise, the consultation itself can take many forms, from a phone call to a full family gathering with an ethics committee, or a retrospective discussion, where cases are reviewed following the consult [29]. Because ethics consultations often involve multiple interactions over time rather than being a single event, CEC using multiple methods are common. [29] Publications addressing any of these types of consultations will be included.

#### Types of outcomes

##### Primary outcomes

A primary outcome of this phase will be a comprehensive listing of potential measures and domains of measurement (i.e., clusters of related measures) for CEC. When considering measures and domains of CEC quality, we will follow the Donabedian Quality-of-Care Framework, which categorizes quality measures as assessing structures, processes, or outcomes [31]. This will not be the first time domains of quality for an aspect of health care have been developed from an examination of themes across a body of literature. This project was inspired in part by the rise of quality improvement studies in health care, where the Donabedian framework is frequently used to categorize measures of health care quality into structural, process, and outcome measures. These categories can also be used to inform the development of quality measures [31]. Reviewing themes across a body of literature is a particularly appropriate method for examining complex interventions in health care. For example, the Institute of Medicine (IOM) has used this same process to develop policy guidelines addressing what is and is not high-quality health care [32, 33]. Likewise, several members of our research team have used this sort of step-wise thematic analysis to develop articles, policy documents, and analytical instruments for assessing other aspects of health care quality [19, 34–37].

Some examples of possible structure, process, and outcome measures for CEC are as follows:

- Structural measures of quality assess such things as institutional, provider, community, and client characteristics: for CEC, these might include measures of CEC accessibility (e.g., on-call availability), consultant training, committee composition, and staff support. [28, 29]
- Process measures of quality assess procedural aspects of service delivery: for CEC, these might include measures related to methods of service delivery, number and type of interpersonal interactions, timing of service delivery, and communication methods used.
- Outcome measures of quality assess clinical, financial, experiential, or other expected end-products of receiving care: for CEC, these might include patient satisfaction, conflict resolution, clinician satisfaction, clinician learning, resource utilization, and changes in staff moral distress or feelings of support [38–47].

##### Secondary outcomes

As secondary outcomes, barriers and facilitators to improved quality of CEC noted in the literature will also be compiled.

#### Information sources and search methods

Ethics case consultations are complex interventions intended to help address complex problems, and there may be multiple components described in a given study with contextual features dependent on factors including study site and the perspectives of various involved stakeholders. For this reason, our scoping review will be very broadly inclusive. We will use the systematic and rigorous Cochrane Collaboration standards, with one noted exception: we will not use a trial-specific sensitive search strategy [24]. This decision was based on our intent to obtain the most comprehensive dataset possible to make effect size comparisons in phase III, but it also serves the purpose of providing a broad collection of literature for our phase II qualitative synthesis. There has been some argument in the literature about the challenges of searching for qualitative literature [48, 49], so we may adopt a more iterative process for that portion, as described in phase II methods below.

To collect papers for inclusion, the authors will develop a search strategy in tandem with an experienced librarian (BH) using multiple thesaurus terms, keywords, MESH terms, and appropriate truncations. A second, unaffiliated librarian will validate this strategy using the Peer Review of Electronic Search Strategies (PRESS) protocol. [50] A draft Ovid MEDLINE search strategy is included (see Additional file 1). The systematic search strategy will begin with just two key terms: (1) clinical ethics and (2) consultation. These are necessarily broad to accomplish a proper scoping review. All references will be uploaded into EndNote Reference Manager (EndNote X7, Thomson Reuters, New York, NY, USA) and Distiller SR (DSR) (Evidence Partners Incorporated, Ottawa, Canada), where duplicates will be removed and recorded for Preferred Reporting Items for Systematic Reviews and Meta-Analyses (PRISMA) reporting [51–54]. A PRISMA-P checklist for this protocol can be found in Additional file 2.

#### Electronic searches

We will use databases in medical science (Ovid MEDLINE, Web of Science, EMBASE, Cochrane Collaboration, Joanna Briggs Institute), ethics (ETHXWeb, JSTOR, Euroethics, Endebit), law (LexisNexis, Westlaw International, Legal Information Reference Center), arts and social sciences (Psychology and Behavioral Sciences Collection, Campbell Collaboration, PsychINFO, ERIC), and grey literature (Academic Search Premier, ProQuest, Conference Proceedings Citation Index-Science, Open Grey, PsychEXTRA, EAGLE, NTIS NYAM Grey, Google Scholar, Google). All databases will be searched from their inception without date restrictions. There will be no geographic restrictions. Articles not in English will be excluded from this review due to expense of translation fees. Articles without full text or without available abstracts will be excluded if they cannot be obtained from the authors following three email attempts. Unpublished doctoral theses will be excluded.

#### Searching other resources

Two authors (LL, AM) will search a number of other sources to further add to the literature for the scoping review. Hand searching of key onsite journals (being those that show up repeatedly in the electronic searches, such as the Journal of Clinical Ethics, Healthcare Ethics Forum, Hastings Center Reports, and the American Journal of Bioethics) and pertinent book reference lists will also be performed per Cochrane standards [24]. Electronic searches of conference proceedings (e.g., International Conference on Clinical Ethics Consultation) and professional organizations (e.g., American Society for Bioethics and Humanities) will be performed to further add to the literature pool. References and citations of all included studies will be searched for other potential literature.

#### Study records and data management

Literature search results will be saved in EndNote. Collected literature will then be exported to DSR and Nvivo11 (QSR International Pty Ltd., Doncaster, Australia).

For the textual analysis, two types of software will be used:

- The qualitative data analysis software Nvivo11 will be used to evaluate textual information from the quantitative studies.
- DSR, where title/abstract screening forms will be used to calculate kappa. DSR may also be used to create extraction forms, which will be used to note demographic and numeric values and for performing quality assessment for phase III.

#### Selection of studies

To select articles for inclusion, an electronic form will be built in DSR, which will serve as a checklist of our inclusion/exclusion criteria for initial title/abstract screening and full-text screening. Two reviewers (LL, RC) will independently review a random sample of 50 articles using the level 1 inclusion/exclusion criteria form in DSR. Reviewers will not be blinded to any portion of the article as this has been shown to have little effect in protecting against bias [55]. Inter-rater agreement for study inclusion will be calculated using kappa statistics and percent agreement. This process will be repeated until the reviewers have a minimum of 90 % agreement. If issues or questions arise about the inclusion/exclusion form, these will be discussed with the other authors. If corrections are made, another round of calibration will be performed until at least 90 % agreement is obtained on the final form. Once there is 90 % agreement on a form, LL and RC will begin independent screening, with ongoing calculation of a kappa statistic. Inclusion criteria will be deliberately broad simply asking “Does this article pertain to CEC quality measurement,” with responses being yes (include), no (exclude), and don’t know (include). For the full text review phase, due to the expected volume, the process will differ. All authors will meet to review a set of full text articles for inclusion where we will use the same question as the title/abstract screening, but only yes (include) and no (exclude) will be the possible responses. This process will be repeated until at least 90 % agreement is obtained. Following that, the reviewers (RC, AM, LL) will meet to repeat the calibration exercise until 90 % agreement is obtained between them. Following those two calibration steps, each reviewer (RC, AM, LL) will independently review articles. If there are issues or hesitations, they will be brought to the larger group for discussion.

#### Assessment of risk of bias in included studies

For the scoping review, no assessment of bias or quality appraisal will be made. Studies will not be excluded on the basis of quality as the scoping review phase is about categorizing the literature, not evaluating it or performing aggregative synthesis.

#### Data synthesis

The literature will be broadly categorized as to whether it addresses quality measurement, including structure, process or outcome measurement, barriers or facilitators to effective consultation, etc. as well as general article information such as study design, country of origin, and other descriptors that might help future researchers access and use this literature. The product of this synthesis will be a written summary with multiple tables and accompanying text of all the literature in the scoping review.

This product will then be brought to a diverse group of stakeholders for review. This review phase will help to assure the relevance of the summary report to the field-at-large. For this, we will present our findings at regional and national conferences, including a statewide group of ethics consultants, including ethicists, lawyers, chaplains, and other end-users. In presenting the results, we will ask about the applicability, pertinence, and potential impact of the findings. This informal stakeholder review process will help inform the direction of the subsequent mixed-method synthesis.

### Phase II (qualitative synthesis) methods

#### Eligibility criteria

Because a comprehensive search will be executed for the scoping review in phase I, the search criteria will not differ. All qualitative studies found in the scoping review will be used for the phase II qualitative synthesis.

#### Search methods for identification of studies

No additional searching of the literature beyond that found in the phase I search will be carried out except in the unlikely event that theory saturation (i.e., the point where the addition of new articles does not add new explanatory theories) is not obtained in the course of the data synthesis described below. This is highly unlikely given the large number of studies anticipated to be found in the initial search (Fig. 1).

#### Study records and data management

Three reviewers (LL, RC, AM) will perform an analysis on included studies using the qualitative data analysis software Nvivo11s to develop a thematic synthesis of the literature, which is described in more detail in the “Data Synthesis” section below.

#### Selection of studies

All articles will have been categorized during the phase I scoping review; all those categorized as qualitative articles pertaining to the measurement of quality of CEC will be included in the phase II qualitative analysis. No exclusions on the basis of quality will be made—articles that may have poorly documented rigor may have contextually rich detail that contributes to the overall thematic analysis.

#### Assessment of risk of bias in included studies

No assessment of quality will be made during phase II. The issue of quality appraisal of qualitative articles in systematic reviews is highly debated [56]. While there is a current trend toward quality assessment, we did not feel it was appropriate for this review, where relatively few empirical studies are expected, where much of the work comprises essays, and where we are more concerned with the views presented than their scientific rigor.

#### Data synthesis

This will follow the iterative approach of a thematic analysis [22, 23] where multiple ideas and conclusions across a body of literature are summarized into key themes, which are then refined into categories and sub-themes. This will proceed in three overlapping stages:

1. Three reviewers (LL, RC, AM) will perform in vivo coding (i.e., using direct quotes using the authors’ original language) of key quotes, metaphors, and ideas from 30 articles selected at random. This will facilitate the creation of an initial codebook of directly extracted material. Using this codebook, all of the included articles will be coded with standard coding strategies in Nvivo11, highlighting concepts and theories emphasized in the articles [57, 58].
2. The initial codebook will be analyzed for themes, which will provide an organizing structure for related areas. Initially, this will be done by further sub-coding in Nvivo11. Comparisons of the groupings will be performed by constant comparative analysis: main theme from article A will be compared to article B, etc. This will form a list of descriptive themes, which will be the starting place for the next stage.
3. The descriptive themes from the above will be further assessed to develop a set of “analytical” themes, which will be the final product and description of the quality domains of CEC. Methods used in previous research by the authors may be used to assist in analysis, including grouping and clustering, vote counting, conceptual mapping, and other qualitative analysis techniques. [22]. These final product “analytical” themes will be used to further elaborate on the quantitative synthesis and scoping review.

These analytical themes will be presented in table format with supporting text. This “summary of findings” will include the number of articles supporting each analytic theme.

### Phase III (quantitative synthesis) methods

#### Eligibility criteria

Because a comprehensive search will be executed for the scoping review in phase I, the initial search criteria (population, intervention, outcomes, etc.) will not differ. Instead, randomized-controlled trials (RCTs) found in the scoping review will be used for the quantitative synthesis. Non-randomized controlled trials, prospective cohort, and quasi-experimental studies will also be included, since there are very few RCTs, but only if their outcomes and methods are sufficiently similar to those in the RCTs. Subgroup analysis will be performed on any non-RCT studies included to show different results according to study type.

#### Search methods for identification of studies

As noted, the scoping review search strategy is far more comprehensive than a RCT-specific search would be and includes both quantitative and qualitative studies. In piloting electronic search strategies, we found that articles reporting on clinical trials were captured using our broader search strategy as well as by the Cochrane highly sensitive search strategy for identifying randomized trials [24]. For that reason, no specific additional literature searches will be carried out for phase III as the entire search is considered to be complete.

#### Study records and data management

Initially, reviewers will perform a further in-depth analysis using several extraction tools:

- Distiller SR, where extraction forms will be used to note demographic and numeric values as well as for performing quality assessment.
- Nvivo11, where textual data will be extracted.
- JMP (JMP®, Version 12. SAS Institute Inc., Cary, NC, 1989-2015.), or another statistical analysis software, will be used for the meta-analysis.

#### Selection of studies

All articles will be classified and categorized in the phase I scoping review. For phase III, all studies from phase I that were categorized as RCTs that report on measured outcomes of CEC will be included. All non-quantitative studies will be excluded. For mixed-method studies, the quantitative component may be used if it is determined to be comparable to a RCT. Trial studies that are not RCTs may be included if they measure similar clinical outcomes as the RCTs.

#### Assessment of risk of bias in included studies

Study quality will be assessed using the Critical Appraisal Skills Programme (CASP) questions [59]. Two reviewers (LL, AM) will independently evaluate studies using the CASP checklist. Where disagreements in any of the evaluations exist, reviewers will consult with content experts (JA, MW, JG, KF) for resolution.

Next, the Grades of Recommendation, Assessment, Development, and Evaluation (GRADE) approach from the Cochrane Collaboration will also be used to assess included studies [24]. This will also be built into a checklist for evaluation of the five areas of (1) risk of bias, (2) inconsistency, (3) indirectness, (4) imprecision, and (5) publication bias [24, 60]. Confidence will be judged as high, moderate, low, or very low. Exclusions based on quality will be made following Cochrane standards [24].

#### Assessment of reporting biases

If sufficient studies are found to perform a meta-analysis, funnel plots will be used to assess for publication bias. Reported outcomes and comparisons will be matched to the study protocol, where available, to assess for reporting bias.

#### Measure of treatment effect

The types of data handled for the phase II synthesis will follow Cochrane standards [24].

- Continuous data
  When the same scales/units are used, mean differences will be calculated. When differing scales/units are used, standardized mean differences will be used. These will be reported with 95 % confidence intervals.
- Dichotomous data
  Risk ratios will be calculated for dichotomous (binary) data. These will be reported with 95 % confidence intervals. If it is more appropriate, odds ratios may be converted to standardized mean differences. Where appropriate results from different trials will be combined.
- Ordinal data
  Ordinal outcomes will be treated as continuous variables or dichotomous variables, depending on thresholds.
- Count data
  For events experienced between two groups that use count data, rate ratios will be calculated.

#### Unit of analysis issues

For RCTs and other studies where randomization was used, ICC estimates will help in examining design effect where the variance may be inflated accordingly. If ICC is not readily available, authors may be contacted. Otherwise, trials may be analyzed using imputed ICC estimates from similar trials and where sensitivity analysis can be performed. In order to account for clustering, authors may be contacted to expand on how causal treatment effect and organizational clustering were separated.

#### Dealing with missing data

For quantitative studies, when study data are missing, authors will be contacted. All missing data will be recorded for later reporting if study authors cannot provide missing data (or if they do not respond). The quantity and patterns, as well as the handling of missing data, will be recorded. Sensitivity analysis may be performed to assess the impact of missing data, which will be addressed in any summary of findings.

#### Assessment of heterogeneity

Heterogeneity of trial studies will be assessed by percentage of total variation (*I*^2^ statistic). Heterogeneity will be assessed using forest plots, chi^2^ (*P* < 0.10), and *I*^2^ tests (low is <49 %, moderate is 50 to 74 %, high is 75 to 100 %). If there is moderate heterogeneity (chi^2^*P* < 0.10 AND *I*^2^ moderate-to high), we will use a random-effects model [24]. If there is no statistical heterogeneity (low *I*^2^), we will combine results using a fixed-effect model.

#### Subgroup analysis and investigation of heterogeneity

Using an *I*^2^ statistic, heterogeneity across subgroups will be analyzed. If data allow, and if it seems helpful in illuminating relationships, a multivariate meta-regression model based on outcomes may be performed.

#### Sensitivity analysis

If components of a study give disproportionate influence to the review, sensitivity analyses will be performed and will be reported in a “summary of findings” table.

#### Presentation of data

A “summary of findings” table will be presented for the quantitative synthesis as well as a forest plot, if possible. There will also be textual and visual depictions of findings to help explain the findings generated from the literature.

A comprehensive list of the steps of the synthesis will be reported either in text or as an appendix for comparison to this protocol. A PRISMA checklist and diagram will be provided to compare to the PRIMSA-P checklist provided in appendix to this article (see Additional file 2) [51–54].

## Discussion

The literature on CEC is wide ranging and includes both quantitative and qualitative work. Relatively little of this literature addresses quality measurement explicitly, but a rather large number of articles and reports implicitly describe issues related to CEC quality and how to measure it.

We have developed a protocol to accomplish a formal systematic review of this diverse literature that will list possible quality measures for CEC found in the included articles, develop categories for these measures (i.e., quality domains) comprising an analytical structure for measuring the quality of CEC, and assess the effects of CEC on clinical outcomes. This mixed-methods review will fill a major gap, as there have been no formal efforts to analyze and synthesize the complete literature on CEC to seek latent consensus around potential quality measures and domains. The protocol we have described will apply a quality assessment lens to this diverse literature, which will contribute to the evolution of theories about quality measurement for CEC and, more importantly, might stimulate the development and dissemination of useful CEC quality measures.

## Abbreviations

ASBH, The American Society for Bioethics and Humanities; CASP, Critical Appraisal Skills Programme; CEC, Clinical Ethics Consultation; DSR, Distiller SR; GRADE, Grades of Recommendation, Assessment, Development, and Evaluation; IOM, Institute of Medicine; PRESS, Peer Review of Electronic Search Strategies; PRISMA, Preferred Reporting Items for Systematic Reviews and Meta-Analyses; PRISMA-P, Preferred Reporting Items for Systematic Reviews and Meta-Analyses Protocols; TJC, The Joint Commission

## Additional files

Additional file 1: Sample Ovid MEDLINE Search. (PDF 42 kb) Additional file 2: PRISMA-P (Preferred Reporting Items for Systematic review and Meta-Analysis Protocols) 2015 checklist: recommended items to address in a systematic review protocol. (DOC 81 kb)
