# Supplementary material for: Domains of quality for clinical ethics case consultation: a mixed-method systematic review
Source: Syst Rev. 2016 Jun 7;5:95. doi: 10.1186/s13643-016-0273-x (PMC4897931; doi:10.1186/s13643-016-0273-x)
Supplement: Additional file 1: — Sample Ovid MEDLINE Search. (PDF 42 kb) [file 13643_2016_273_MOESM1_ESM.pdf]

Additional file 1. Sample Ovid MEDLINE Search

Ovid Medline	Search executed 7/6/2015 results 6209
Concepts	Ethics Consult
Free-text words	((bioethic* or ethic*) adj3 (consult* or dialogue* or discussion* or talk* or session* or meeting* or examination* or service* or counsel* or mediat*) or ((clinic* or medical or healthcare or health care or hospital*) adj4 (ethic* committee* or bioethic* committee*))).tw,kf. or
MeSH	ethics consultation/ or ethics committees, clinical/
